# Visible Social Interactions Do Not Support the Development of False Belief Understanding in the Absence of Linguistic Input: Evidence from Deaf Adult Homesigners

**DOI:** 10.3389/fpsyg.2017.00837

**Published:** 2017-06-02

**Authors:** Deanna L. Gagne, Marie Coppola

**Affiliations:** ^1^Department of Psychological Sciences, University of Connecticut, StorrsCT, United States; ^2^Department of Linguistics, University of Connecticut, StorrsCT, United States

**Keywords:** theory of mind (ToM), visual perspective taking, false photograph, deafness, homesign, false belief task, Nicaraguan Sign Language

## Abstract

Congenitally deaf individuals exhibit enhanced visuospatial abilities relative to normally hearing individuals. An early example is the increased sensitivity of deaf signers to stimuli in the visual periphery (Neville and Lawson, 1987a). While these enhancements are robust and extend across a number of visual and spatial skills, they seem not to extend to other domains which could potentially build on these enhancements. For example, congenitally deaf children, in the absence of adequate language exposure and acquisition, do not develop typical social cognition skills as measured by traditional Theory of Mind tasks. These delays/deficits occur despite their presumed lifetime use of visuo-perceptual abilities to infer the intentions and behaviors of others (e.g., Pyers and Senghas, 2009; O’Reilly et al., 2014). In a series of studies, we explore the limits on the plasticity of visually based socio-cognitive abilities, from perspective taking to Theory of Mind/False Belief, in rarely studied individuals: deaf *adults* who have not acquired a conventional language (Homesigners). We compared Homesigners’ performance to that of two other understudied groups in the same culture: Deaf signers of an emerging language (Cohort 1 of Nicaraguan Sign Language), and hearing speakers of Spanish with minimal schooling. We found that homesigners performed equivalently to both comparison groups with respect to several visual socio-cognitive abilities: Perspective Taking (Levels 1 and 2), adapted from Masangkay et al. (1974), and the False Photograph task, adapted from Leslie and Thaiss (1992). However, a lifetime of visuo-perceptual experiences (observing the behavior and interactions of others) did *not* support success on False Belief tasks, even when linguistic demands were minimized. Participants in the comparison groups outperformed the Homesigners, but did not universally pass the False Belief tasks. Our results suggest that while some of the social development achievements of young typically developing children may be dissociable from their linguistic experiences, language and/or educational experiences clearly scaffolds the transition into False Belief understanding. The lack of experience using a shared language cannot be overcome, even with the benefit of many years of observing others’ behaviors and the potential neural reorganization and visuospatial enhancements resulting from deafness.

## Introduction

Congenitally deaf individuals can exhibit enhanced visual perception and visuospatial abilities that reflect neural reorganization in response to an altered sensory landscape and/or experience using a natural sign language in the visual modality, such as American Sign Language (ASL). Examples of skills demonstrating such enhancement in deaf individuals include attention to motion in the periphery (e.g., Neville and Lawson, 1987b), mental rotation (Emmorey et al., 1998), and face processing (McCullough and Emmorey, 1997). An extensive literature has documented this phenomenon and has also begun to discern the relative contributions of deafness and language experience to the locus and nature of the subsequent neural reorganization (e.g., Neville and Lawson, 1987a; Emmorey et al., 1993, 1998; Bavelier et al., 2001; Codina et al., 2011). Recent work has uncovered more details regarding the associations between experiential factors such as type and timing of language exposure, and the corresponding reorganization of specific neural areas. For example, Cardin et al. (2013) showed that changes in regions of the left superior temporal cortex (STC) in deaf individuals can be attributed to exposure to sign language, while plasticity in the right STC results from their altered sensory landscape (namely, a lack of auditory input). In this special issue, Codina et al. (2017) report differences in peripheral visual sensitivity due to differential sensory experiences (deafness vs. hearing) and the timing of acquisition of sign language (early vs. later).

However, these enhancements due to a lack of auditory input or use of a visual language do not necessarily extend to other cognitive domains. For example, although deaf children and adults spend their entire lives observing the behaviors of others and using this visual information to navigate social interactions, the ability to predict others’ beliefs, desires, and subsequent behaviors, commonly known as Theory of Mind (ToM) (e.g., Premack and Woodruff, 1978; Baron-Cohen, 2001; Call and Tomasello, 2008, among many others), is more strongly associated with the quality and amount of language input, and the age of exposure to such input (e.g., de Villiers and Pyers, 2002; Schick et al., 2007; Howard et al., 2008; Meristo et al., 2012).

Wellman and Liu (2004) and Peterson et al. (2005) present a scaling of precursor *mental* (belief, desire, and emotion) abilities to ToM abilities, that is, an ordering of tasks assessing knowledge and understanding of mental states that are necessary for children to succeed on false belief tasks. Here we focus on the contribution of *visual* experiences to the development of these social cognitive skills, and report a similar scaling of visuospatial abilities that appear to be prerequisites for social cognitive abilities. This investigation is particularly motivated by our interest in the development of socio-cognitive abilities among rarely studied individuals who do not have access to language but who nevertheless have rich visuo-social experiences, and is informed by arguments such as those proposed by Dijksterhuis and Bargh (2001), who present a theoretical link between gains in perception and subsequent gains in social cognition.

**FIGURE 1:**
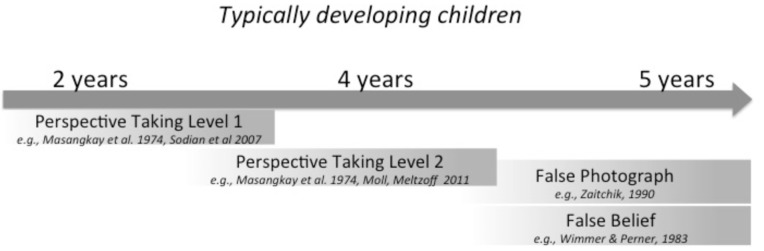
**Development of precursor abilities to False Belief along a Theory of Mind (ToM) developmental trajectory in typically developing, hearing children.** In these studies, perceptual experience, language experience, social experience, and biological maturation are tightly intertwined (e.g., Bigelow and Dugas, 2009), and continues to be intertwined as ToM develops into adulthood, for instance, in the use of figurative language such as sarcasm (e.g., Happé, 1994; O’Reilly et al., 2014). Our goal in the present paper is to determine which, if any, of these precursor abilities can develop on the basis of visual experience alone (i.e., which are robust given a lack of access to language and the richer social experiences afforded by language).

The series of studies we report here is designed to discern the contribution of visuospatial perceptual experiences to the development of a sequence of skills pertinent to social cognition (**Figure 1**). All of these abilities have been identified as possible precursors to False Belief understanding, the hallmark measure of a mature ToM in typically developing children (e.g., Flavell et al., 1981; Zaitchik, 1990; Sodian et al., 2007; Moll and Meltzoff, 2011). Of course, other abilities have also been identified as precursors to ToM (e.g., joint attention, specific syntactic constructions, etc.). In the present study we focus on those precursor abilities that (a) are visually based and (b) can be assessed using tasks that require minimal linguistic demands in terms of instructions and responses.

In the majority of previous studies, the perceptual experiences, language experiences, educational experiences, and biological maturation of participants are all tightly intertwined and presumably synergistic. One way to attempt to disentangle these factors is to look to populations whose experiences vary systematically in particular ways. One such population is deaf children: approximately 5–10% of deaf children are born to deaf signing parents (Mitchell and Karchmer, 2004), the remaining 90–95% have hearing parents who do not know sign language. Only a small percentage of these hearing parents become fluent users of sign language (Meadow-Orlans et al., 2003), and the resulting variability in deaf children’s language experiences offers a window into the contribution of early language exposure to these precursor abilities. However, prior work examining these specific skills has tested deaf children in the context of an educational or early intervention setting, whether it be focused on spoken language, sign language, or both modalities. Thus, most deaf children in prior studies have been exposed to at least *some* language, intervention, and education (e.g., Peterson and Siegal, 1998; Meristo et al., 2007; Shield et al., 2016).

Our goal in the present paper is to determine which, if any, of these precursor abilities are robust in the face of a lack of access to language and education, especially given arguments that exposure to an established visual language whose structure capitalizes on spatial perspective taking may scaffold socio-cognitive abilities (Courtin, 2000). The rarely studied groups in the current work offer a unique opportunity to identify the relative contributions of visual experiences alone. When we say “alone” here, we refer to the unusual situation faced by homesigners (described in more detail below). That is, studying homesigners offers a way to assess the limits of visual experiences in driving socio-cognitive skills in the absence of a language model, participation in a shared linguistic community, and rich educational experiences. To that end, we asked participants to (a) indicate *what* (image) another person sees (Perspective Taking Level 1); (b) indicate *how* another person sees [a 3-D object (Perspective Taking Level 2; both tasks adapted from Masangkay et al. (1974)]; and (c) recall a visual reality that does not match the current visual reality [False Photograph, (e.g., Zaitchik, 1990)].

These specific tasks were chosen for several reasons. First, they are well-known precursors to False Belief success along the social cognition continuum. That is, typically developing children can often succeed on these tasks before or at about the same time they are able to succeed on False Belief tasks (**Figure 1**). Second, their focus on visual information (**Table 1**) is well suited to our question of how far the use of visual information can take an individual on the developmental path to ToM. Third, they minimize linguistic tasks demands relative to tasks that rely on storytelling (e.g., the Sally-Anne Task, Baron-Cohen et al., 1985), use particular linguistic structures (e.g., de Villiers and de Villiers, 2000) or specific mental-state vocabulary (e.g., Howard et al., 2008). Two facts motivated our examination of these abilities in adult homesigners: (1) these tasks have not been done with deaf children (regardless of language delay); and (2) these tasks have never been done with homesigners. Here we will see whether reducing the linguistic load in the task might reveal explicit ToM understanding with this population.

**Table 1:** Summary of the requirements of each task in the current study.

Task demands	PTL1	PTL2	Mental rotation	False photo	Experiential false belief
Is response about Identity or Orientation of object?	Identity	Orientation	Orientation	Identity	Identity
Content of representation: Visual or Mental	Visual	Visual	Visual	Visual	Mental
Is representational conflict Within Self or Self vs. Other?	Self vs. Other	Self vs. Other	Within self	Within self	Self vs. Other

*Note that Mental Rotation [as tested by Shield et al. (2016) was not tested here, but is provided for the sake of completeness]. PTL1 = perspective taking level 1, PTL2 = perspective taking level 2.*

### Participant Groups

For our purposes, Homesigners are a group with little to no exposure to conventional linguistic input, little to no educational experience (**Table 2**), but lifelong experience using a visual communicative system. However, homesigners’ experiences differ from those of the “atypical” child and adult participants typically studied in the domain of ToM in at least two additional ways. Homesigners do not participate in a linguistic community, and they have scant experience with formal education. Therefore, we included two comparison groups to better discern the contributions of each of these experiences, and help focus our attention on the role played by visual experiences: Deaf signers of the first cohort of Nicaraguan Sign Language (NSL) and hearing Nicaraguans who have not had formal education. We now describe each of these participant groups, and explain how their particular characteristics serve our goal of narrowing down the potential explanations for performance on tasks measuring various abilities in the domain of ToM.

**Table 2:** Summary of participant groups and characteristics.

Group (n)	Mean age (range)	Age at first language exposure (range)	Educational experience (mean, range)
Homesigners *n* = 4 (3 male)	31.5 years (26–35 years)	*N/A*	0.5 years (0–1.5 years)
NSL Cohort 1 *n* = 6 (3 male)	41 years (35–45 years)	3.84 (2–5 years)	10.5 years (6–13 years)
Unschooled Hearing Spanish Speakers n = 7^∗^ or 8 (5 male)	33.25 years (19–56 years)	From birth	0.4 years (0–3 years)

*^∗^One Unschooled Spanish Speaker did not participate in the False Photograph task.*

#### Homesigners

Homesigners are deaf individuals who have not acquired either a spoken or sign language; their deafness precludes adequate access to the spoken language around them (e.g., Goldin-Meadow, 2003). In Nicaragua, homesigners’ family and community environments do not include opportunities to access sign language (Polich, 2005). They are raised by hearing families who do not sign, and their geographical and/or economic circumstances preclude access to special education and/or the signing Deaf community. Despite this lack of access to linguistic input, they develop their own gesture systems called *homesign* (Coppola, 2002; Coppola and Newport, 2005). In developing countries like Nicaragua, few resources exist for identification, intervention, and education of children with disabilities. An extremely small proportion of the deaf population participates in the recently emerged Deaf community who uses NSL (Labato, 2017). Thus, it is not uncommon for many deaf individuals to reach adulthood without benefiting from the crucial language exposure provided by participating in a community of deaf signing individuals.

Despite their lack of conventional linguistic input, *homesigners* in Nicaragua continue to use their gesture systems as their primary means of communication into adulthood. They show no signs of social impairment or inhibition, readily engaging socially with familiar and unfamiliar individuals, and exhibit none of the social impairments/difficulties found in individuals with autism. Thus, they enjoy relatively typical social interactions with their hearing families, friends, and neighbors, with the caveat that these hearing communication partners do not fully share the homesign system with the deaf individual in their family (Carrigan and Coppola, 2017). While homesign is not a fully developed language, mature homesign systems exhibit a range of linguistic properties found in fully developed languages, such as morphophonological regularities (Brentari et al., 2012), morphosyntactic structure (e.g., Coppola et al., 2013), and the grammatical relation of subject (Coppola and Newport, 2005).

Finally, homesigners provide us with an attractive population to explore these areas of interest particularly because they are biologically more mature than the deaf children in most previous studies of this nature. In the absence of language, and the consequent reduced opportunities to take others’ perspectives (visual or otherwise), we might expect a protracted trajectory of ToM development. By exploring how ToM abilities do or do not emerge on the path to adulthood given these unusual circumstances, we hope to contribute to the current understanding of ToM development.

#### Nicaraguan Sign Language: An Emerging Language

Nicaraguan Sign Language is an indigenous sign language that emerged from a newly expanded center for special education in Managua during the late l970s to early 1980s (Polich, 2005). The first group of signers is known as “Cohort 1”; these signers initially formed the Deaf community and began creating the language through their interactions with each other at the center for special education. Like Homesigners, Cohort 1 signers of NSL did not have access to linguistic input transmitted from any pre-existing language model. However, Cohort 1 signers did engage in language genesis with their peers (e.g., Senghas, 2003; Senghas et al., 2005). NSL signers of all cohorts (Cohort 1 and the subsequent children who entered the school later, representing Cohorts 2, 3, and so on) interact with many other users who use the system as a primary language, i.e., members of the Deaf community in Managua, and are thus part of a linguistic community. Homesigners, by comparison, use their gesture systems with hearing people their entire lives – hearing people who only use these gestures with the homesigner and never with each other.

Another significant difference between Homesigners and Cohort 1 signers is the fact that Cohort 1’s (and subsequent cohorts’) introduction to the linguistic community is situated within an educational or vocational context (Polich, 2005; Senghas et al., 2005). As it is for most deaf children born to parents who do not already know a sign language, it is the schools that provide *both* educational and primary linguistic experiences through peer interactions. This will be explained further in the context of the next group, the Unschooled hearing Spanish Speakers.

Thus, the main motivations for including Cohort 1 signers as a comparison group are: (1) to investigate two deaf populations in the same cultural context whose language experiences differ minimally; and (2) to establish an anchor point using a previously studied group whose false belief abilities had previously been studied using this methodology.

#### Unschooled Nicaraguan Spanish Speakers

As mentioned, like most deaf children born to hearing parents who do not know a sign language, the vast majority of NSL signers in past and present studies gained access to their linguistic community via educational settings (Polich, 2005). We therefore cannot separate having a linguistic community from education in either NSL signers, who have both, or Homesigners, who have neither. Unschooled hearing Spanish Speakers have had a complementary set of experiences to Cohort 1 signers: they have full access to an established language and a language community from birth, but have little to no education (**Table 2**).

The reasons that the Unschooled Spanish Speakers did not go to school are straightforward and are unlikely to reflect an uncontrolled selection bias: five of the eight unschooled hearing participants were full-time agricultural workers; the other three worked for their family businesses, making and selling food products in their local communities. Their lack of education primarily resulted from economic restrictions and distance to the nearest school.

## Study 1: Perspective Taking Level 1

We began by assessing all participants on a simple baseline visual perspective task. Based on results from their “mountain task,” Piaget and Inhelder (1956) theorized that children could not understand the visual perspective of others until well into childhood, after about the age of 9. Subsequent research has shown that young children do understand others’ perspectives, though this ability is acquired incrementally, with the ability to understand *what* someone else sees (Perspective Taking Level 1) available earlier in development than the understanding of *how* that person sees it (Perspective Taking Level 2) (e.g., Masangkay et al., 1974; Flavell et al., 1981).

By the age of 3 typically developing children can easily answer the *what* question, as measured by verbal tasks [e.g., “what does the experimenter see?” (e.g., Masangkay et al., 1974)] or by the second year of life as measured by looking (Luo and Baillargeon, 2007; Sodian et al., 2007) or assisting (Moll and Tomasello, 2006) behavior.

No published studies have reported on Perspective Taking Level 1 abilities in deaf children^1^. Given previous studies with typically developing children, which confound maturation, language exposure, and possibly educational experiences, we cannot make a clear prediction for the homesigners’ performance on Perspective Taking Level 1 tasks.

### Method

All procedures for all studies reported were approved under University of Connecticut IRB #H10-306. All participants provided written informed consent to participate in this study, and those identified in the images of the manuscript provided written informed consent for their publication.

### Participants

The 4 Nicaraguan Homesigners, the 6 Cohort 1 NSL users, and the 8 Unschooled Spanish Speakers described in **Table 1** participated in this study.

### Materials

**FIGURE 2:**
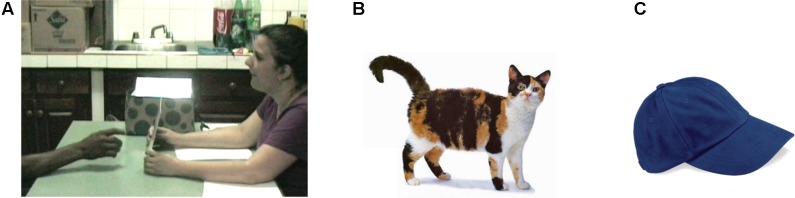
**(A)** The experimenter (right) and a participant (left) engaged in the Perspective Taking Level 1 task. He sees one image, (**B**, “cat” or **C**, “cap”), while the experimenter could see the other. All images were photographs of real objects and not line drawings.

Level 1 perspective taking abilities were tested using two-dimensional stimuli, namely eight images of common objects, animals, and humans presented on 8½” × 11” laminated sheets (see **Figure 2** for examples of Cat/Cap). All the images chosen were familiar items in Nicaragua, varying in category from humans to animals to inanimate tools, and have been used successfully with all of these participant groups (Richie and Yang, 2013).

### Procedure

Each round used two images from the previously mentioned list, arranged into four pairs: Cat/Cap, Wheelbarrow/Fishing Rod, Cow/Girl and Pitcher/Chicken. First, both images were shown to the participants, and participants were asked to identify the objects. This was done for two reasons – first, it familiarized the participants with the images, and second, it created common referential expressions between the participant and the experimenter (particularly important because homesigners each have their own idiosyncratic gesture systems).

The participant and experimenter sat across from each other at a table (**Figure 2A**). For each of the aforementioned sets, after familiarization and naming of the images, the two images were placed back to back, with one image facing the participant and the other facing the experimenter. The experimenter then asked, using the appropriate communication system (gesture/homesign, NSL, or Spanish): “What do *you* see?” and “What do *I* [the experimenter] see?” (**Table 3**). Both perspective questions were asked for each set, and each set of images was flipped so that the participant had an opportunity to see every image. Feedback was provided as needed during the first pair of images, Cat/Cap, to clarify instructions.

**Table 3:** Sample interaction for Cat/Cap.

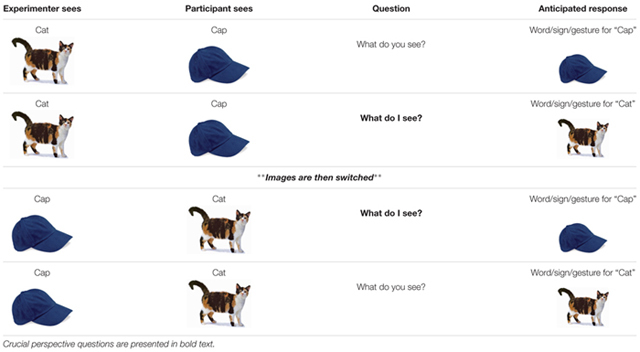

### Results and Discussion

Participants in all groups answered the control questions correctly (“what do *you* see?”) and correctly named the image that the experimenter saw during test trials; that is, all participants performed at ceiling. Keep in mind that the homesigners in this study have had *extremely limited* experience with a conventional sign language; however, this lack of linguistic input and community did not have a negative impact on their performance. Therefore, if language is required in order to succeed on this task, their idiosyncratic visual and gestural experiences must exceed the threshold. The fact that the groups differ dramatically in their language experiences, but not in their performance, seems to suggest that simple maturation and/or life experience can support success.

## Study 2: Perspective Taking Level 2

Our next question investigates whether the homesigners understand *how* someone else sees an object – that is, do they understand that when they and another person are looking at the same object from different angles, that the other person’s perspective is different from their own, and what that other perspective on that object would look like? We address this in the next study, with a task investigating Perspective Taking Level 2 (Masangkay et al., 1974; Flavell et al., 1981). Previous studies have shown that Level 2 perspective taking abilities are available later in development than Perspective Taking Level 1 (e.g., Masangkay et al., 1974; Flavell et al., 1981), though Perspective Taking Level 2 may be available to children as young as 36 months of age (Moll and Meltzoff, 2011).

As it was for Perspective taking Level 1, few studies have directly measured Level 2 Perspective Taking in deaf populations. In a study of natively signing children with and without Autism Spectrum Disorders (ASD), Shield et al. (2016) found differences in results for Level 2 perspective taking: native-signing children with ASD did not perform as well on Level 2 tasks as did the typically developing native-signing Deaf children. However, the children with ASD did not show this difficulty with mental rotation tasks, which consider different perspectives of the same object, but without considering another person’s perspective of the object (**Table 1**).

There has also been some debate in the literature as to whether exposure to and use of sign language and the spatial perspective taking inherent in using a spatial language boosts perspective taking abilities, which may in turn boost ToM development (Courtin, 2000). However, more recent studies suggest that using a visual language confers no benefit to signing children or to subsequent ToM development (Courtin and Melot, 2005; Shield et al., 2016). Therefore, we hypothesized that there would be no differences between the groups’ success on Perspective Taking Level 2 tasks.

### Method

The participants for this study were the 4 Nicaraguan Homesigners, the 6 Cohort 1 NSL users, and the 8 Unschooled Spanish Speakers shown in **Table 2**.^2^

### Materials

**FIGURE 3:**
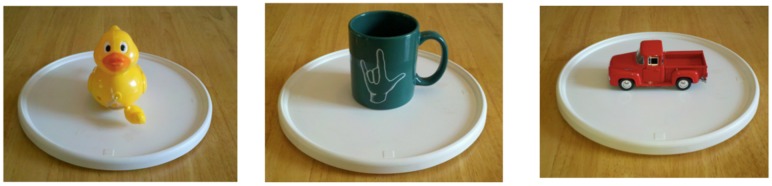
**Photographs of the three objects used in Perspective Taking Level 2: Duck, Mug, and Truck**.

Level 2 perspective taking abilities were tested using a procedure adapted from Reed and Peterson (1990) using minimal language/gesture with the homesigners and three-dimensional objects (e.g., Shield et al., 2016). Three familiar objects were included in our task: a toy duck, a mug with identifiable sides (a hand design on one side and a handle), and a toy truck (**Figure 3**).

### Procedure

**FIGURE 4:**
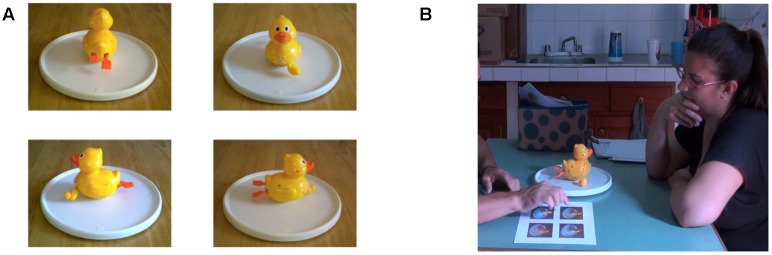
**Sample answer array showing the four perspectives tested in Perspective Taking Level 2 (A)**, and the experimenter observing a participant choosing a perspective from the array **(B)**.

Each object was presented to the participant on a turntable so that it could be rotated easily either by the experimenter or the participant. Testing was done with the turntable on a surface between the participant and the experimenter. Front/back perspectives were tested first with all objects, then side (left/right) views were tested. As in Level 1, participants were asked, using appropriate communication methods, “What do *you* see?” and “What do *I* [the experimenter] see?” (**Table 4**). Participants were given an 8” × 11” laminated sheet displaying the four possible perspectives of the object being tested (**Figure 4**). Participants could respond either by selecting the correct image or by describing the correct perspective (e.g., “You see the back of the duck” or “You see the duck’s feet.”), however, the experimenter encouraged participants to indicate the correct image whenever possible for clarity in coding and for consistency across participant groups, particularly for the homesigners, who often solely relied on selecting images rather than describing the experimenter’s perspective. The experimenter repeated the question for insufficiently descriptive responses such as “You see the duck.” The duck was used for familiarization with the task, and feedback was provided during duck trials. By the end of the familiarization/practice trials, all participants save for two (one homesigner and one hearing Spanish speaker) correctly responded at 100% to the *experimenter’s* view for all four perspectives on the duck, as measured by the last time each of these four perspectives was tested (front, back, left side, and right side). These two participants are discussed further in the “Results and Discussion.”

**Table 4:** Sample interaction for Front/Back trials of object: duck.

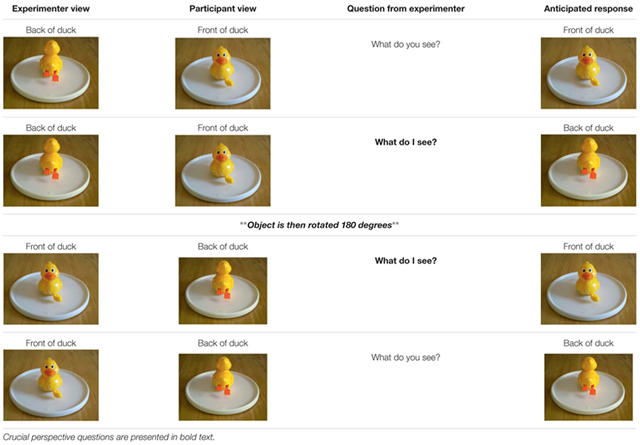

**Table 5:** Performance by individual participant across both experiential false belief conditions.

	Homesigners				Cohort 1						Unschooled Spanish speakers							
Participant	1	2	3	4	1	2	3	4	5	6	1	2	3	4	5	6	7	8
Appearance/Reality					✓	✓	✓				✓	✓	✓	✓				
Unexpected contents					✓						✓	✓	✓					

*No participant passed the Unexpected Contents condition without also passing the Appearance/Reality condition.*

### Results

**FIGURE 5:**
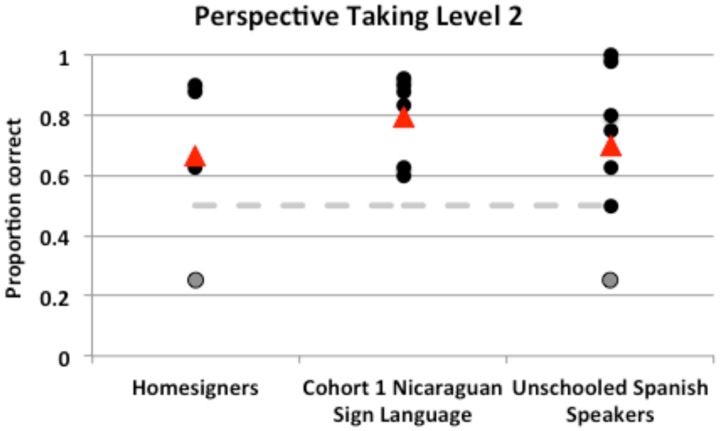
**Participant groups did not differ in Perspective Taking Level 2 scores, and the mean score of every group was above chance.** Individual scores are represented by black/gray circles, and group means by triangles. All but three participants significantly above chance (the critical value, 0.50) is indicated by the dashed line. The two participants who did not score at ceiling by the end of the practice trials are represented by gray circles.

Level 2 Perspective Taking (three-dimensional objects) was coded for accuracy across test trials (Mug and Truck). Only the first response to questions asking about the *experimenter’s* perspective are reported (i.e., the “What do I see” questions for the two objects *mug* and *truck*, totaling four trials). Because no significant differences were found between Left/Right and Front/Back scores across participants via a paired-samples *t*-test, (Left/right *M* = 0.76, *SD* = 0.21; Front/Back *M* = 0.71, *SD* = 0.3), *t*(16) = 0.84, *p* = 0.413, we report a single overall score per participant (**Figure 5**).

All group means were above chance (0.25); indeed, all but two participants scored at or above chance. Groups did not differ in their performance [Kruskal–Wallis non-parametric test, *H*(2) = 0.44, *p* = 0.802] with a mean of 8.8 for homesigners, 10.7 for NSL Cohort 1, and 9 for the Unschooled Spanish Speakers^3^.

### Discussion

We asked whether homesigners, whose language exposure and educational experiences are very limited, can correctly predict another person’s visual perspective of the same object from a different angle. When compared to individuals who have language exposure from birth (the Unschooled Spanish Speakers), or other deaf individuals who have had educational experiences (Cohort 1 NSL signers), we found no differences in overall performance.

The one published study that has investigated Perspective Taking Level 2 in deaf individuals found differences between deaf individuals with and without ASD (Shield et al., 2016). The current findings contribute to that small body of work, affirming that Shield et al.’s (2016) finding was likely due to a social deficit in the deaf individuals with ASD, rather than to a language-specific deficit. Both of the current analyses contribute to this interpretation: (1) the fact that homesigners, despite their lack of exposure to linguistic input, performed above chance on the Perspective Taking Level 2 task; and (2) the lack of difference among the groups, who differed markedly in their language experiences, on the Perspective Taking Level 2 task.

Although we found no differences among groups, we did find interesting results in two individuals – one Homesigner and one Unschooled Spanish speaker^4^, indicated by gray circles in **Figure 5**. While the presence of their scores did not affect the overall results, we wish to address two points about these participants. First, their scores on the test items parallel those of their practice items; that is, they were the only two participants to not achieve ceiling on the experimenter’s perspective by the end of the practice items. However, they did reach ceiling for *their own* perspective (“What do you see?”), showing that their difficulty with the task was likely not due to any inability to interpret the three-dimensional object to two-dimensional images in the answer array^5^.

Second, their incorrect answers for the experimenter’s perspective in the test trials seem to indicate egocentric perspectives – 80% of the Homesigner’s responses and 60% of the Unschooled Spanish Speaker’s incorrect responses were the image of their own perspective, rather than the other two potentially incorrect choices in the 4-picture array. If they had chosen the other potentially incorrect choices, one could argue that they understood that the experimenter could not possibly see the same perspective as they, but they just could not mentalize or translate that perspective to the image(s). Because their responses were so strongly egocentric, we must conclude that they likely do struggle with understanding other people’s perspectives.

We also observed an interesting strategy employed by some Unschooled Spanish Speakers that was not observed in the other two groups. Namely, they used language to talk their way to the correct answer out loud by verbalizing their own perspective (e.g., “I see the driver’s side…”), and then coming to conclusions about the experimenter’s perspective (e.g., “… so you must see the passenger side.”). Employing this strategy did not elevate this group’s scores as compared to the other two groups.

Although the homesigners superficially scored similarly to the other two groups in this visual perspective taking task, it is possible that the quality or depth of their understanding of others’ *visual* states does not translate to an understanding of *mental* states. We hypothesize that the underlying quality of their visual social interactions with others are different, *given their different linguistic experiences* (namely, that they are severely restricted, such as playing hide-and-seek *without* the benefit of phrases like “where are you? I can’t see/find you!”). Another main difference is that while they *interact* daily with their families and friends, these communicative interactions do not rely on a shared linguistic system. We propose that this lack of a shared linguistic system is the likely cause for their difficulties with more advanced ToM tasks, even when those tasks do not strongly rely on language in their procedure or instructions.

## Study 3: False Photograph

In order to test participants’ ability to maintain a previous reality in the face of changes to the present reality, we conducted a “false photograph” task. Pioneered by Zaitchik (1990), the False Photograph task closely imitates False Belief tasks. However, instead of requiring participants to consider another’s belief, it requires them to consider a previous reality that does not represent the current state of affairs.

Zaitchik’s (1990) original study and others since then (e.g., de Villiers and Pyers, 2001) suggested that typically developing children struggle with false photograph tasks just as much as they do with false belief tasks. This pattern suggests that the difficulty with false beliefs for typically developing children may not be in the belief as much as in the conflict between the prior knowledge and current state of reality. Some groups of children show a different pattern, calling this interpretation into question. Children with autism, and deaf children with hearing, non-signing parents (who do not have the early benefit of accessible language), reliably pass false photograph tasks while struggling with false belief tasks (e.g., Leslie and Thaiss, 1992; Peterson and Siegal, 1998; de Villiers and Pyers, 2001). This difference in patterning may point to a benefit of language – the children with autism were minimally able to communicate (in order to participate in these tasks), and the deaf children had access (albeit late) to either spoken or signed language. However, the children with autism or hearing loss were, at minimum, 2 years older than the typically developing children studied in False Photograph tasks. This age difference suggests that given enough developmental time, even without the benefit of language, false photograph understanding may develop in any individual. Working with homesigners – individuals who have *never* had the benefit of learning from linguistic input, or engaging in a shared language environment, can help us disentangle these factors.

### Participants

The participants for this study were the 4 Nicaraguan Homesigners, the 6 Cohort 1 NSL users, and 7 of the 8 Unschooled Spanish Speakers represented in **Table 2**.^6^

### Procedure

The procedure for the current False Photograph task was modeled after the Identity-Change Photograph^7^ task in Leslie and Thaiss (1992), also used with deaf children in de Villiers and Pyers (2001). Participants were first presented with a Fujifilm Instax 210 camera (similar to a classic Polaroid camera whose prints develop within minutes), and three objects: a doll, a duck, and a truck. All participants were first asked to name the three objects. This was mainly so that the experimenter would be familiar with the specific gesture used by each homesigner to name each object, since these gestures could vary across homesigners.

First, as a control question, the doll was placed on a chair and a picture was taken. The developing photograph was then placed face-down on a table and the participants were asked what the photograph would show. All participants correctly stated that the image would be that of the doll. Then the doll on the chair was replaced by the duck and another photograph was taken. The new developing image was placed, again, face-down on a nearby table, within sight of the participant. Before the participant was asked about the new photograph, however, the duck was then replaced by a toy truck and the participant was finally asked what image would appear on the face-down photo.

### Results and Discussion

All participants in all groups were able to correctly state that the face-down polaroid image would be of the duck that previously occupied the chair, and not the toy truck that was currently in that location.

Typically, False Photograph tasks are reported along with results from False Belief tasks, which we will address in the general discussion. It is interesting to note, however, that the age of success for typically developing children on False Photograph tasks is similar to the age of success for False Belief tasks – again, an age at which their language has developed quite a bit. This would typically lead one to believe that False Photograph success may also be associated with a certain amount of language exposure. However, the homesigners, who have not acquired any established language, spoken or signed, succeeded on this task; this finding supports and strengthens previous findings (e.g., Peterson and Siegal, 1998) showing that success on False Photograph tasks may be dissociable from language experience. Interestingly, given the degree and duration of the homesigners’ lack of exposure to language and education, we show that this ability may develop from life experience alone.

## Study 4: Experiential False Belief

Thus far we have explored various visually based precursor abilities that have previously been related to False Belief success, and have shown that homesigners succeed on all of these tasks: Study 1 (Perspective Taking Level 1), Study 2 (Perspective Taking Level 2), and Study 3 (False Photograph) (see **Figure 1**). Homesigners’ success on Perspective Taking Level 2 shows their ability to understand and consider another person’s visual perspective of an object (e.g., Masangkay et al., 1974; Flavell et al., 1981). With respect to the relative timing of these precursor abilities, typically developing hearing children pass False Photograph and False Belief tasks at around the same point in development (Zaitchik, 1990). On the basis of this set of findings, one might predict that adult homesigners would also succeed on False Belief.

A number of previous studies have shown poorer performance on FB tasks among deaf individuals than their normally hearing peers. The consensus of these studies is that the relatively poor performance of deaf individuals does not result from the experience of being deaf itself, but from the consequent delay in language exposure (Peterson and Siegal, 1999; Rhys-Jones and Ellis, 2000; Woolfe et al., 2002; Courtin and Melot, 2005; Moeller and Schick, 2006; Morgan and Kegl, 2006; Schick et al., 2007; Meristo et al., 2012, among others). What these studies have shown is that ToM abilities are *delayed* commensurate with the degree of delay of exposure to sign language. But what if the child is *never* exposed to an established language? Does ToM never progress past a certain point? What if that child creates a system of communication themselves? Is that system enough to scaffold FB success? Most previous tests of false belief (e.g., Peterson et al., 2005; Peterson and Slaughter, 2006; Schick et al., 2007) relied on linguistically conveying a sequence of events and then explicitly asking the child a critical question such as “Where will Sally look for the marble?” [from the classic Sally-Anne task in Baron-Cohen et al. (1985)]. Less linguistic means of conveying the story events, including, for example, thought bubbles (Morgan and Kegl, 2006) or sequenced pictures (Pyers and Senghas, 2009) still rely on participants’ experience with literacy conventions that is typically only gained in formal education settings. Due to their relative lack of formal education, homesigners have extremely limited experience with books, stories presented as a sequence of pictures, or other sophisticated literacy conventions. Thus the current minimally linguistic, experiential approach gives us a means of probing homesigners’ FB understanding that avoids these issues.

In line with previous studies showing a link between language experience and FB performance, Pyers and Senghas (2009) also found an effect of using a still-emerging language on FB success, comparing the FB performance of successive cohorts of signers of NSL. Both Cohort 1 and Cohort 2 signers in their study entered the signing community before the age of 6 years; in this way they are similar to the deaf children in the studies described above whose access to language is delayed. However, it is worth noting two differences: the NSL signers were tested as adults, and each cohort of signers entered a very different situation with respect to the type of language available in their environment, as we elaborate below.

Cohort 1 signers were the first creators of what is now NSL, and are from the same cohort of NSL signers as those in the current studies; Cohort 2 signers are the individuals who entered the Deaf signing community after Cohort 1, and who learned NSL from them. Pyers and Senghas (2009) found that Cohort 1 signers, who used fewer mental verbs when describing videos depicting belief or desire events, succeeded less frequently on False Belief tasks than the Cohort 2 signers, who used more mental verbs in their descriptions of those events^8^. Note that the Cohort 2 signers are the first in the Nicaraguan Deaf community to benefit from a language model, namely, the signing of the Cohort 1 signers who preceded them in the center for special education (vertical input)^9^. While they did not have language models, Cohort 1 signers benefited from peer-to-peer interactions (horizontal input) in the context of a linguistic community. The individual homesigners who are the focus of the current studies lack such a linguistic community. Thus, comparing the homesigners and Cohort 1 signers will reveal the contribution of Cohort 1’s shared emerging language to FB success.

Despite scant access to language input and formal education, homesigners show remarkable abilities to access and express information. As noted earlier, they create relatively complex gesture systems that display structure at various levels of linguistic analysis (e.g., Coppola and Newport, 2005). They also spend their entire lives observing the behavior of the people around them, relying solely on the visual information accessible to them. Therefore, we ask: can life experiences apart from language, that is, visuo-social experiences, provide enough information about others’ thoughts, beliefs and desires to scaffold ToM development in the absence of linguistic input?

### Participants

The participants were those described in **Table 2**: 4 Homesigners, 6 NSL Cohort 1 signers, and 8 Unschooled Spanish Speaking adults.

### Procedure

In order to minimize effects of having a shared language and/or educational experiences (such as those found when using picture-completion tasks), we employed an *experiential* False Belief task developed by Pyers (2005). Instead of conveying a narrative using language, or relying on literacy conventions, this methodology uses the participant’s own personal experiences in the course of the task to establish the false belief situation (*Experience Phase*). Then, in the *Prediction Phase*, the participant is asked to make predictions about another person’s behavior (choices). Both phases impose minimal productive and receptive communication demands on the participant. We now describe the procedure in detail.

**FIGURE 6:**
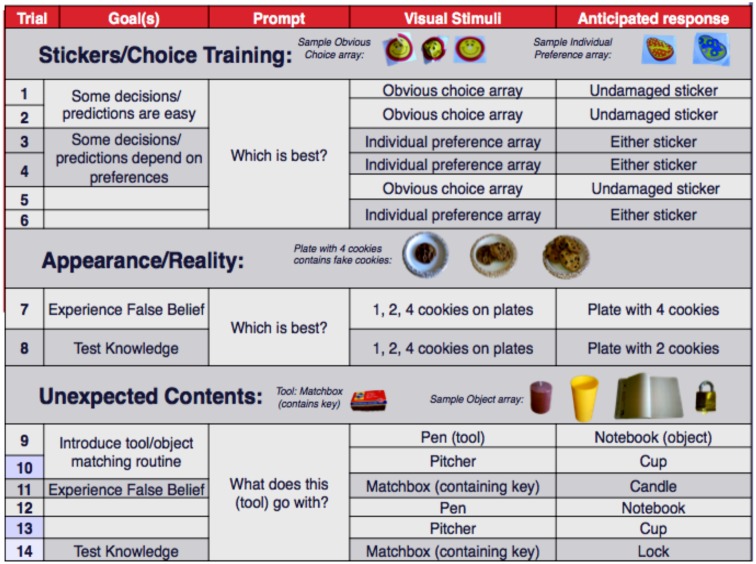
**The phases and individual trials that each participant saw, once as an experiencer and then once as a predictor of a confederate’s choices.** All relevant ordering possibilities were counterbalanced.

As described above, each participant was given first-hand *experiences* with Appearance-Reality (A/R) and Unexpected Contents (UC) false belief situations. They then participated in a *prediction* game in which they earned an incentive for making correct predictions. The procedure is described in great detail because the incremental, implicit understanding of the task instructions, and how participants should respond, are essential to our commitment to a minimally verbal procedure that fairly assesses the ToM abilities of homesigners in particular. **Figure 6** summarizes the 14 trials that each participant saw, first in the *Experience* phase, and then again in the *Prediction* phase.

#### Phase 1: Participant as Experiencer

##### Part 1: Control/training: Stickers

All participants first engaged with six sticker trials of two types: “obvious choice” or “individual preference” (3 of each, totaling 6 trials). As an *experiencer* this phase familiarized participants with the process of choosing items from an array, and (non-verbally) demonstrated that a choice on a particular trial may be obvious (e.g., three stickers bearing identical images in which one is pristine, and the other two are crumpled or ripped), or that a choice might be based on one’s preference (e.g., two different-colored smiley face stickers) (**Figure 6**). The sticker trials also ensured that participants understood that they had to choose only one element of the array on each trial.

##### Part 2: Appearance/Reality False Belief

In the A/R *experiencer* phase, the participant saw three plates holding one, two, and four cookies. Unbeknownst to the participant, the four “cookies” were very convincing ceramic composite replicas. The experimenter encouraged each participant to indicate the “best” plate. For the homesigners, this was done by pointing at the participant, indicating the three plates of cookies, followed by a thumbs-up, thumbs-down gesture combined with a questioning look. All participants in all groups chose the plate with four cookies during their experience phase. After selecting this plate, they were then encouraged to try a cookie from that plate, at which point they discovered that the cookies were not real. The cookies were then returned to their original locations and the question or gestures were repeated, this time to check the participant’s knowledge that the plate only appeared to contain four “cookies,” and that the plate containing two cookies should be considered the “best.” At this point, all participants in all groups selected the plate with two cookies, demonstrating that they successfully internalized their false belief. That is, they understood that their previous belief that the four cookies on the plate were real was in fact, a false belief.

##### Part 3: Unexpected contents false belief

**FIGURE 7:**
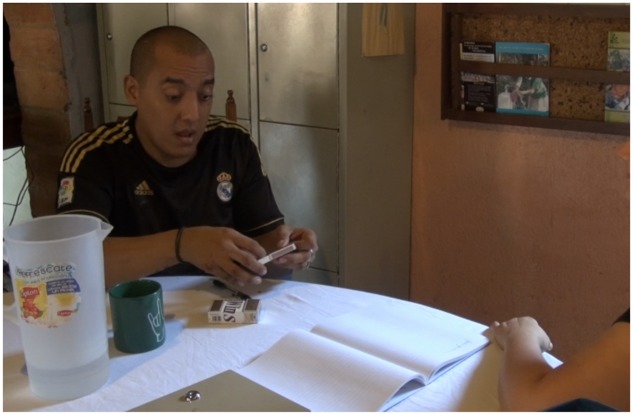
**A participant engaged in the Unexpected Contents portion of the Experiencer Phase.** He has just opened the matchbox after having matched it with the cigarettes, and has discovered that the matchbox actually contains a key, not matches.

In the UC *experiencer* condition, the participant was shown one of the following arrays of four real objects: (1) a sheet of paper, a glass, a small padlock, and a candle; or (2) a notebook, a mug, a lockbox, and a box of cigarettes (**Figure 6**). The participant was then presented with a series of tools and was asked to indicate which object in the array each tool is used with. First, the participant was presented with a pen, and was asked to match it with an object. The correct choices were the paper (first array described above) or notebook (second array described above); the order of arrays was counterbalanced across participants. Upon choosing the paper/notebook, the participant was asked to make a mark on the paper/notebook. This was done for two reasons: first to show that the pen was functional, and second to establish a routine of “using” the tool presented with the object it was used with. Second, the participant was presented with a pitcher of water and asked to match it to its object (the correct choice being the glass or mug). Again, upon choosing, the participant was asked to pour water into the vessel (**Figure 7**). Third, the participant was presented with a matchbox (which, unbeknownst to the participant, contained a key, but no matches), and again was asked to match it to one of the objects. Note that at this point there are two objects in the array that have not been matched to a tool: one that could be lit with a match (candle or cigarettes) and one that was seemingly unrelated (a small padlock or a lockbox). As an *experiencer*, the first correct response should be based on the external appearance of the matchbox, and thus the correct match would be the object that could be lit: either the candle or the cigarettes. Upon choosing, the participant was encouraged to light the candle or a cigarette, and subsequently discovered that the matchbox contained a key, not matches (**Figure 7**).

The objects in the array were then switched out for their functional equivalents [i.e., array (1) to (2), that is, paper to notebook, etc.]. However, the three tools (pen, pitcher, and matchbox) were left in view of the participant, so he or she could see that no one, including the experimenter, touched them during this changeover. The entirety of the UC condition was repeated, now with the participant knowing what was in the matchbox, matching tools to their objects. At this point, the key trial is the matchbox trial, during which the participant should demonstrate his or her knowledge that the matchbox contained a key (instead of matches), and should therefore be matched with the padlock/lockbox, and not the candle/cigarettes.

### Phase 2: Participant as Predictor (Confederate as Experiencer)

**FIGURE 8:**
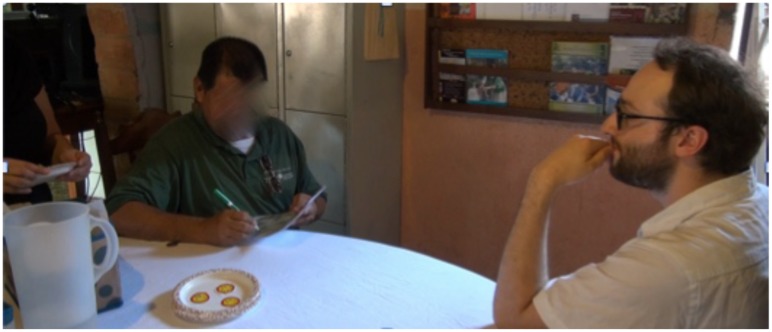
**Participant (left) engaged in the Sticker portion of the *Prediction Phase* of the experiment.** Here he is indicating on the laminated sheet which sticker (of two damaged, one pristine) he predicts the confederate (right) will choose.

To reiterate, after experiencing all 14 trials of the task, and more importantly, after directly experiencing the two false beliefs (the four objects on the plate only *appear* to be cookies, and the matchbox contains a key and not matches), each participant participated in all of the trials again, but this time as a *predictor* of another person’s choices (**Figure 8**). The confederate whose choices were predicted by the participant was a member of the research team who had not previously participated in any aspect of this task with the current participant and who had remained out of sight of the participant and experimenter for the duration of the task up until this point. Moreover, the experimenter invited the participant to collude with the experimenter by emphasizing that the other person who would be brought in (the confederate) had “not seen” the game before. Finally, for each trial, the participant indicated the item he or she thought the confederate would choose *before* the confederate actually made a selection, by marking a set of laminated sheets depicting each array of objects.

### Part 1: Control: Sticker Trials

In the *prediction* phase, sticker trials served to implicitly instruct the participants that: (1) sometimes it is easy to predict someone else’s behavior (obvious choice trials), (2) sometimes it is harder (individual preference trials), and (3) correct predictions earn a small monetary reward [5 Córdobas per correct prediction (max. 70 Córdobas or US$2.75)] across all trials (a healthy incentive given typical local incomes).

Sticker trials also gave participants the opportunity to show their understanding that individual preferences might vary and/or encouraged participants to consider the other person’s preferences in cases where the participant predicted that the confederate would choose the same sticker that they themselves had chosen in the experiencer phase. All participants experienced one or both of two possible outcomes: (1) making an incorrect choice when *predicting* the confederate’s choice of sticker. In this case their subsequent failure to earn the incentive emphasized that they should consider the other person’s preferences. In outcome (2), participants predicted sticker preferences for the confederate that *differed* from their own, showing that they indeed were considering the other person’s preferences and not just going with what they (the participant) themselves had chosen previously as an experiencer.

Finally, the sticker trials served to train participants how to indicate their predictions of the confederate’s choices by marking the laminated sheet displaying the array of objects associated with each trial. This was particularly important for the two groups who did not have educational experiences (the Homesigners and Unschooled Spanish Speakers, many of whom are not literate), and who may not have many opportunities to interact with images in this way or to use writing tools (dry erase marker). No participant displayed any difficulty in using these items during the task – they could indicate their choices by circling, making dots, or marking lines on the images they chose, and all participants showed their understanding of the task procedure during the sticker trials.

Note that while each Experiencer and Predictor phase began with sticker trials, the order of A/R and UC False Belief trials was counterbalanced across participants, as well as across the Experiencer and Predictor phases of the experiment for each participant.

### Part 2: Appearance/Reality False Belief

In the A/R *prediction* phase, the participant observed the experimenter ask the confederate “which is [the] best [plate of cookies]?” Before the confederate made his choice, however, the participant was asked to indicate which image on the laminated sheet (one, two, or four cookies) the confederate would choose (out of sight of the confederate, of course). Then the confederate made his choice of four cookies as the “best.” Recall that the confederate at this point is acting as a naïve participant who should not know that the four cookies are fake – he should have a false belief given the appearance of the cookies – the same false belief the participant just experienced themselves. The participant is then told whether he/she was correct in his/her prediction and rewarded (or not). Just as in the participant’s version of the experiencer phase, the confederate is then told to take a cookie from the plate he chose, at which point he “realizes” that the four cookies are fake. The fake cookie is returned to the plate and the knowledge question is posed to the confederate, with the participant predicting the confederate’s current knowledge of the fake cookies, and earning the monetary incentive for the correct knowledge prediction. All participants in all groups correctly predicted that the confederate would choose the plate with two cookies at this point, regardless of whether their initial prediction was correct.

### Part 3: Unexpected Contents False Belief

In the *prediction* phase of UC, the participants were again asked to predict how the confederate would match the tools to the objects in the array before them by indicating their prediction on the laminated sheet for each trial, with correct predictions earning incentive payments. Recall that the third tool (a matchbox containing a key) is the crucial match – the confederate continued the naïve act and first matched the matchbox based on the appearance of the matchbox – that it ought to have matches and thus be used with the item that could be lit (the candle or cigarettes). The participant was then told whether their prediction for the confederate’s choice was correct, earning the appropriate reward for correct predictions. Importantly, the participant ought to have recalled their own false belief and predicted the candle or cigarettes. Regardless of the participant’s correct/incorrect prediction, the confederate was then asked to proceed and “use” the matches to light the candle or cigarettes, at which point the confederate “realizes” that the matchbox contains a key and no matches. The array is switched out, as it was for the participant’s experiencer phase, and the confederate is then asked the knowledge question(s), going through all three tools, with the participant making predictions now “knowing” that the confederate has realized what is in the matchbox, and earning the appropriate incentives through the remaining trials.

### Results

**FIGURE 9:**
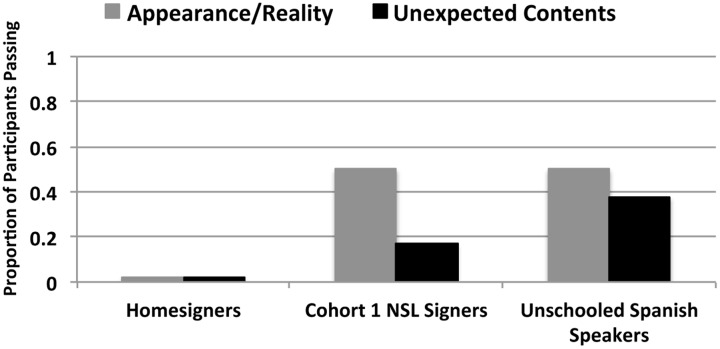
**Proportion of participants in each group who passed the Appearance-Reality condition (gray bars) and Unexpected Contents condition (black bars).** The Unschooled Spanish Speakers, who had access to an established language from birth (but who did not have educational experiences) did not perform at ceiling. Cohort 1 Nicaraguan Sign Language (NSL) Signers performed similarly to previous reports. The lack of passing among the Homesigners and moderate success of NSL Signers suggests that access to language, even an emerging one like NSL, promotes False Belief success.

In this task, participants were asked to correctly predict a confederate’s choices across a variety of preference trials and tool-object matching trials. The crucial questions were an A/R condition (one, two, or four cookies, with the plate of four cookies containing fake cookies) and an UC condition in which a box of matches actually contained a key. For the A/R condition, a participant passed if they initially (that is, before the confederate realized that the four cookies were fake) predicted that the confederate would choose the plate containing four “cookies.” Participants failed this task if they initially predicted that the confederate would choose the plate containing two (real) cookies. For the UC condition, a participant passed if they initially predicted that the confederate would match the matchbox to the item to be lit (i.e., the cigarettes or the candle), and failed if they predicted that the confederate would match the matchbox to the item that needed a key (i.e., the padlock or the lockbox). None of the Homesigners, who lack a linguistic community, passed; however, immersion in a linguistic community did not guarantee passing for NSL signers and Unschooled Spanish Speakers. In sum, for the A/R condition, no homesigner passed, 3 of the 6 NSL signers passed (50%), and 4 of the 8 Unschooled Spanish Speakers passed (50%). For the UC condition, no homesigner passed, 1 of the 6 NSL signers passed (17%), and 3 of the 8 Unschooled Spanish Speakers passed (37%) (**Figure 9** and **Table 5**).

### Discussion

This experiential false belief study is the first to be conducted with a rarely studied population: homesigners, individuals free of congenital social impairments whose linguistic input is extremely limited. Despite generating gesture systems featuring a surprising degree of linguistic complexity, many years of communicative engagement with family members and friends, and relatively typical social and vocational experiences, none of the four adult homesigners passed either of the experiential false belief tasks. We now address (and reject) alternative explanations and interpretations of this poor performance.

#### Alternative Explanations and Interpretations

##### Task issues

One potential objection is that homesigners performed poorly because the task design was too difficult for anyone to succeed. The fact that 1/6 NSL signers and 3/8 Unschooled Spanish Speakers scored 100% (that is, succeeded on *both* the A/R and UC questions) demonstrates that the task is passable. A second possibility is that the homesigners did not understand the task. We are confident that the incremental nature of the task design, and the gradual escalation of task demands, ensured that homesigners understood what was expected of them. Below we argue that the homesigners did not respond randomly, that they in fact systematically performed worse than chance, and that their patterns of responding are interpretable given our theoretical framework.

Two patterns in the data indicate that the homesigners (and participants in other groups who we report as “not succeeding” at the A/R or UC false belief questions) did not choose answers at random. First, these participants always chose the answer that they themselves knew to be the true state of the world. For example, in the A/R cookie trials, no one predicted that the confederate would prefer the plate with one cookie; everyone who responded in error predicted the plate with two cookie (the plate with the largest number of “real” cookies). Similarly, no participant (who answered incorrectly) predicted that the confederate would pair the matchbox with anything but the lock or lockbox, an even more striking “error” because superficially, matchboxes do not pair with things that lock. They then gave these same responses when asked the true belief question after seeing the confederate experience a false belief.

Participants’ overall better performance on A/R trials supports the validity of the task. Recall that the order of false belief trial types was counterbalanced such that half of the participants received the A/R trial first, and half received the UC trials first. Those participants who made an error on their first false belief trial had the opportunity to learn from their failure to obtain the financial incentive. However, the pattern of results shows that participants in all groups failed to learn from their errors on the false belief trials, suggesting that the task indeed measured their false belief abilities rather than their ability to learn.

##### Memory issues

Memory problems, and not an inability to represent and manipulate the confederate’s mental representation of the world, might account for homesigners’ poor performance. Maybe homesigners forgot their previous experiences with false beliefs in the *Experiencer* phase; in that case, they would have responded in accord with the “face value” of the cookies on the plates and the matchbox and thus should have answered correctly that the plate containing four cookies was best, and that the matchbox matched the candle/cigarettes. Because they consistently chose the “correct wrong answer” – meaning the plate with two cookies (not the plate with one) and the padlock/lockbox (rather than the paper or the mug), they demonstrated that their memory of their previous experiences is intact and is in fact driving their choices.

##### Other methodological considerations

Our extensive experience with the homesigners who participated in this study indicates robust comprehension and production of pointing and communicative intent (Coppola and So, 2005; D’Entremont and Seamans, 2007; Tomasello et al., 2007). Indeed we take advantage of this for all tasks in this study. Thus, the minimal-communication demands of the task are unlikely to be the source of the homesigners’ difficulties. One drawback shared by many extant false belief studies with deaf and hard of hearing participants (including the present study) is that they were run by hearing experimenters. Deaf and hard of hearing children routinely experience that hearing people have information they do not have access to (e.g., Jenny is running down the hall); these cumulative experiences may lead them to generalize/over attribute knowledge to hearing experimenters in these types of tasks (Gagne, 2015).

Given previous studies showing delays in False Belief abilities in deaf children and adults with compromised access to language (e.g., Peterson and Siegal, 1999; de Villiers and Pyers, 2002; Morgan and Kegl, 2006; Schick et al., 2007; Pyers and Senghas, 2009), it is not surprising that the homesigners performed poorly. However, these results add to our understanding that ToM abilities do not emerge in adulthood on their own, as a result of many years of life experiences and social interactions, in the absence of linguistic input and participation in a shared linguistic community. Indeed, these findings highlight the contribution of language experience to the later ToM success of late-language acquiring deaf adults in studies such as Peterson and Wellman (2009) and O’Reilly et al. (2014). These studies all support the following conclusion: experience with language later in development (i.e., after early childhood) helps ToM skills, but it does not universally lead to success for all individuals. Importantly, the current study underscores the finding that a lack of exposure to a linguistic community uniformly results in striking impairments in ToM understanding, even in mature individuals.

#### Malleability of Theory of Mind Abilities in Adulthood

Our results also replicate the results of Pyers (2005), who employed (and innovated) the minimal-language, minimal-communication Experiential False Belief task with signers of NSL from Cohorts 1 and 2. In fact, 5 of the 6 Cohort 1 signers in the current study were the same individuals tested by Pyers (2005). Strikingly, the performance of these 5 individuals did not improve, despite 10 additional years of life experience and social interactions. These results appear to conflict with studies showing that ToM abilities *can* improve later in life. How can we resolve this? One explanation might be that false belief performance relates not only to *the age* at which an individual is exposed to language, but also to the *type* of language they are exposed to (i.e., established or emerging) (Pyers and Senghas, 2009). The O’Reilly et al. (2014) participants were exposed to Australian Sign Language, an established sign language, which already had abundant mental-state verbs in its lexicon. Thus, a main difference between the late-exposed adults in O’Reilly et al. (2014) and the Cohort 1 signers in the current study (who were all exposed to sign by age 5) is their relatively greater access to mental-state verbs such as “believe” and “know.” This lexical richness may have helped to develop the Australian participants’ ToM understanding and growth into adulthood. Pyers and Senghas (2009) argue that the reduced availability of such mental-state verbs in Cohort 1 signing was the limiting factor for development of their false belief abilities. They further argue that increased exposure to those words over a 2-year period improved Cohort 1’s performance, though not to ceiling, and not on all types of false belief tasks.

#### Unschooled Spanish Speakers’ Apparent Difficulties with the Task

Our most surprising result is that the Unschooled Spanish Speakers, who *did* have exposure to an established language from birth, did not universally succeed: only 4 of the 8 participants succeeded on the A/R task, and only 3 of 8 succeeded on UC. One noteworthy pattern is that a greater proportion of the Unschooled Spanish Speakers passed *both* the UC *and* A/R tasks, compared to the NSL signers, leading to higher overall scores. Given our focus on the role of language in the development of ToM abilities, we were initially a bit surprised that the unschooled hearing Spanish speakers did not succeed on all false belief tasks given their full access to an established language. However, recall that while the Spanish speakers, like Cohort 1, do engage others using a shared language (Spanish), they are also like the homesigners in that they do not have educational experiences. Several studies have demonstrated the positive influence of a shared community language on the development of ToM (e.g., Morgan and Kegl, 2006; Pyers and Senghas, 2009), as well as the positive impact of mental-state language (e.g., Howard et al., 2008) or even specific linguistic structures (e.g., de Villiers and de Villiers, 2000). Previous studies have also found that even adults with full access to language and education in westernized cultures do not necessarily perform at ceiling in behavioral ToM tasks requiring explicit responses (e.g., O’Reilly et al., 2014), or even implicit responses (e.g., Senju et al., 2009).

Indeed, studies of children in preliterate cultures show they do not always perform at ceiling (e.g., Avis and Harris, 1991; Vinden, 1996, 1999; Chasiotis et al., 2006) but do show developmental gains between 3 to 8 years of age (e.g., Callaghan et al., 2005). These children also show better performance on A/R tasks than on UC tasks (Vinden, 1996). These results parallel our current findings with the two non-Homesigning groups: participants (including the Unschooled Spanish Speakers) were more likely to pass the A/R task than the UC task, though neither group performed at ceiling.

We also understand the findings of the Unschooled Spanish Speakers in the following context. First, the hearing Spanish speakers in this study spend the majority of their time around familiar people and in familiar contexts. This is, in part, a consequence of how we selected them: we did not want to recruit participants who chose (or whose families chose for them) not go to school because of an endogenous factor (such as mild intellectual disability). Thus the participants in our study came from families where formal schooling was too remote, or deemed not necessary in order to sustain the family. Consequently, participants were from relatively self-sufficient families, and do not regularly interact with a wide variety of others. In this way they are well matched to the social interaction profiles of the homesigners.

Second, and related to the first reason, for both the homesigners and the Spanish speakers, other people tend to fill in gaps in social cognition, and thus these skills are not challenged to develop further. Third and finally, it is difficult to compare the false belief performance of Spanish speakers with no formal schooling to prior research with unschooled *adults*, because none exists that we are aware of (though we would be very happy to learn of it). Previous work has focused exclusively on the abilities of unschooled children (e.g., Avis and Harris, 1991; Vinden, 1996, 1999; Callaghan et al., 2005; Chasiotis et al., 2006, among others). It is important to keep in mind, too, that research with well-educated adults in developed countries also reveals surprising limits on their propensity (but not necessarily capacity) to use ToM skills in appropriate communicative contexts (see, for example, Keysar et al., 2003; Apperly et al., 2010).

Having addressed issues related to the use of the experiential false belief task with these understudied populations and their results and interpretations, we turn now to considering the overall pattern of results from this series of four studies, and their implications for our understanding of the relationship between mental and visual representations of others’ experiences without the contributions of a shared language.

## Overall Discussion

In a series of four developmentally sequenced studies, we investigated the possibility that a lifetime of socio-visual experiences scaffolds socio-cognitive development, particularly in the realm of ToM. We worked with three understudied populations in Nicaragua: (1) Homesigners, deaf individuals who have extremely limited language input and educational experiences, (2) Cohort 1 NSL signers, the first group of deaf individuals to contribute to the creation of a new sign language in Nicaragua, who benefit from both a language community and educational experiences, and (3) Unschooled Spanish Speakers, hearing individuals in Nicaragua who have been exposed to spoken Spanish from birth, but who, like the Homesigners, have very little to no formal educational experience.

Across three tasks previously suggested to be precursor abilities to False Belief success [the gold standard task for measuring mature ToM abilities (e.g., Wimmer and Perner, 1983; Baron-Cohen et al., 1985)], we found that Homesigners either performed at ceiling (Perspective Taking Level 1 and False Photograph), or did not significantly differ from the two comparison groups (Perspective Taking Level 2). However, when presented with an innovative, minimally linguistic, experiential False Belief task (Pyers, 2005) which tested False Beliefs that can be conveyed primarily and effectively in the visual modality (A/R and UC), Homesigners as a group did not succeed (**Table 6**).

**Table 6:** Summary of tasks and the aspects of representational conflict and self/other addressed by each, and performance of each of the groups in the current studies.

Task demands	PTL1	PTL2	Mental rotation	False photo	Experiential false belief
Is response about Identity or Orientation of object?	Identity	Orientation	Orientation	Identity	Identity
Content of representation: Visual or Mental	Visual	Visual	Visual	Visual	Mental
Is representational conflict Within Self or Self vs. Other?	Self vs. Other	Self vs. Other	Within self	Within self	Self vs. Other
**Performance by participant group**		
Homesigners	All groups at ceiling	No group differences in success	*Not tested in current studies*	All groups at ceiling	A/R: 0 UC: 0%
NSL Cohort 1					A/R: 50% UC: 17%
Unschooled Spanish Speakers					A/R: 50% UC: 37%

*PTL1, Perspective Taking Level 1; PTL2, Perspective Taking Level 2; A/R, Appearance/Reality; UC, Unexpected Contents.*

Our results show that Homesigners do not have difficulty in understanding that others may have perspectives of the world that differ from their own (Perspective Taking Levels 1 and 2), and that the current state of the world does not necessarily reflect a previous state (False Photograph). Their understanding of these things is not limited to an understanding of identity, but extends to differing perspectives of the same object (Perspective Taking Level 2). Homesigners’ difficulties in the Experiential False Belief task is not due to problems with memory (they could have passed by “forgetting” their previous experiences and responding solely based on the external appearance of the items in question), or difficulties with understanding others’ desires (they can successfully proceed through the control/sticker trials of the Experiential False Belief task, which asks about desire without presenting a False Belief about the stickers). They also made no incorrect predictions about the “true belief” questions – those that asked about the confederate’s choice after the confederate realized the true state of the world. Thus, we can be confident that the homesigners understand that seeing is knowing, yet may still struggle to understand that not seeing is not knowing (as shown by their difficulty with false belief questions). Taken together, these results demonstrate that visual information, plus potential contributions of maturation, can get one pretty far along the ToM developmental trajectory (**Figure 1**). Importantly, the abilities in question are still limited to the visual realm – this accumulated experience gathering and processing visual information throughout a lifetime does not support the ability to predict another person’s behavior in a false belief context.

### Probing Homesigners’ Lack of False Belief Success

We suggest that the homesigners’ failure to predict others’ behavior is also not due to an inability to apply a meta-representation, at least visually (Smith et al., 2013). However, the limitation may be one of linguistic meta-representation, which is not testable with homesigners, given the communicative structures available to them. Although the precursor abilities we investigated required consideration of another’s mind in that they asked about another person’s perspective (a visual representation), they did not necessarily ask about the content of another person’s belief (a mental representation) (**Table 6**). It may be the case that the relatively greater lexical resources available to the Cohort 1 signers [i.e., mental-state verbs (Pyers and Senghas, 2009)], and to the Unschooled Spanish Speakers contributed to the successes of some participants in those groups. We also acknowledge that success may not be solely based on the *availability* of mental verbs, but the *frequency* of their use (e.g., Howard et al., 2008). We should note that not all Cohort 1 and Unschooled Spanish Speaker participants passed the false belief tasks, which begs the question of possible contributions of education and other language-use contexts that may employ mental-state language frequently. These individuals in Nicaragua (deaf or not) may not engage as often in the types of play that may use the kind of language that contributes to ToM development as we know it in the United States (e.g., “Hide and Seek”).

Perspective Taking is likely a precursor to False Belief success. The current findings suggest that the complete lack of language input (for Homesigners) and the sparse mental language available (to Cohort 1) limits ToM development without hindering the development of Perspective Taking abilities.

### Implicit vs. Explicit Measures

While the Homesigners did not succeed on the Experiential False Belief task we conducted, we cannot necessarily conclude that they are incapable of understanding others’ beliefs. We raise this point given previous studies with typically developing infants who, at the time of testing, also had not had much language or educational experience (e.g., Onishi and Baillargeon, 2005). Perhaps implicit measures of False Belief would be able to detect abilities in the homesigners that are not detectable using the Experiential False Belief task we employed here [e.g., the anticipatory looking task used by Senju et al. (2009)]. Though the current experiential false belief task avoids many of the linguistic and literacy convention demands imposed by the majority of false belief tasks, it does still require the participants to produce an explicit response (marking a prediction on the laminated sheet depicting the array of possible objects). Two previous studies of implicit FB in deaf infants and children show that difficulties in FB may start as early as 17 months (Meristo et al., 2012), and may persist into middle childhood (Meristo et al., 2016). What is left to explore, however, is whether the delay in implicit False Belief prediction found in these deaf infants persists into adulthood. Senju et al. (2009) suggest that for individuals with ASD, difficulties in implicit abilities persist into adulthood, though these same individuals are able to – using their increasing language abilities – overcome difficulties in explicit tasks. The homesigners may prove to be a population with the inverse pattern: language barriers that persist into adulthood but no congenital cognitive deficit to bar the development of implicit ToM abilities.

Positive findings with homesigners using an implicit measure could lend credence to two-system accounts of social cognition (e.g., Apperly and Butterfill, 2009). In these accounts, implicit abilities are available early in life, and possibly shared with non-human animals, but explicit responses are developed later by humans, and may depend on language. This suggestion would align with studies of the neural basis of False Belief and Perspective Taking abilities that show differential activation in the temporo-parietal junction (TPJ)/posterior superior temporal sulcus (pSTS) and the medial prefrontal cortex (MPFC). Aichhorn et al. (2006) suggest that the pSTS/TPJ may be responsible for making “cold” or factual judgments about others’ minds (such as in Perspective Taking, where no behavioral prediction is needed), whereas the MPFC may be employed for behavior prediction. Given the results of our studies, we offer that the development of MPFC calculations of predicted behavior may be language-dependent, whereas the development of pSTS/TPJ computation may be divorced from language (either maturational, or developed from visual or language-independent social interaction). However, this is an empirical question beyond the scope of the current paper.

## Conclusion

In our view, the contribution of the current work is demonstrating that success in visual perspective taking does not automatically or inevitably lead to understanding others’ unseen mental states, despite extensive observations of human interactions. The findings from the four tasks reported here, taken together, fill gaps in the existing literature regarding the relationship between visual socio-cognitive abilities and later (visually based) False Belief success. First, we contribute data on ToM abilities in one population that has not been studied previously (Homesigners), as well as from two highly understudied populations: signers of an emerging language (Cohort 1 NSL signers) and hearing Spanish speakers with little to no educational experiences. We found that all three groups are equally able to succeed on Visual Perspective Taking (Levels 1 and 2) as well as False Photograph, but that they vary in their abilities to succeed at False Belief. Additionally, we showed that while False Belief success may likely be language dependent (even when the task minimizes the need for communication and maximizes firsthand visual experiences), success on Visual Perspective Taking (Levels 1 and 2) and False Photograph tasks is likely independent from language experience. Further studies are needed, most likely involving implicit measures of false belief, to investigate ToM abilities in those who do not succeed at the current False Belief tasks, as well as possible neural correlates for such differential abilities.

## Ethics Statement

University of Connecticut Institutional Review Board Protocol # H10-306. All participants were consented according to an approved process by the University of Connecticut IRB protocol H10-306. Both authors are native American Sign Language signers, are fluent in spoken Spanish and have also had previous extensive contact with the homesigners in this study as well as with the Cohort 1 signers. All signed/gestured consent interactions were videotaped and a simplified consent information sheet was created to accommodate basic reading abilities for Cohort 1 signers and Unschooled Spanish Speakers. Homesigners were consented with a hearing, Spanish speaking relative present who was familiar with homesign to confirm understanding. Cohort 1 participants were usually consented in groups of 2–3, to allow for group questions and clarifications as needed. Because the majority (if not all) of the Unschooled Spanish Speakers had minimal reading abilities, the consent form(s) and information sheet(s) were read aloud to the Spanish speakers with ample opportunity for questions. All participants indicated their agreement to the consent process by printing or signing their name, or making their mark.

## Author Contributions

DG and MC contributed to the stimuli creation, DG collected the data, DG and MC analyzed the data and wrote the manuscript.

## Conflict of Interest Statement

The authors declare that the research was conducted in the absence of any commercial or financial relationships that could be construed as a potential conflict of interest.

## Footnotes

1 Given that the literature contains only one unpublished study [Remmel’s (2003) dissertation] of Perspective Taking Level 1 abilities in deaf children, insufficient basis exists for making a prediction regarding Homesigners’ performance.

2 One Unschooled Spanish Speaker who was tested outside at the farm where he worked did not complete the second half of Level 2 (Left/Right) because the onset of a rainstorm necessitated packing up all of the equipment. We therefore based his overall score on just the first half of the task (Front/Back). Across participants, Left/Right (*M* = 0.76, *SD* = 0.21) and Front/Back (*M* = 0.71, *SD* = 0.3) scores did not differ [*t*(16) = 0.84, *p* = 0.413].

3 This analysis includes the two participants who did not score at ceiling by the end of the practice trials. Removing these participants does not change the non-significant result [*H*(2) = 0.12, *p* = 0.941], with means of 9.2 for homesigners, 8.7 for NSL Cohort 1, and 8.1 for the Unschooled Spanish speakers.

4 Interestingly, these two participants are a mother/son pair, which raises the question of a familial/genetic contribution. Nothing in their everyday behavior suggests that they would not succeed on this relatively straightforward task.

5 This observation has also been made about 4-year-olds by Gzesh and Surber (1985).

6 Due to time constraints, one Unschooled Spanish Speaker did not participate in the False Photograph task.

7 This particular version of the False Photograph task was chosen in anticipation of our False Belief tasks, which do not include a Change-of-Location question (as several False Photograph questions do) but do include Unexpected Contents and Appearance-Reality questions, which more closely relate to an identity question.

8 This finding accords with studies in the United States showing that frequency of mental-state language in children’s input (e.g., Howard et al., 2008) and ability to use mental-state language in complement clause structures (e.g., Hale and Tager-Flusberg, 2003; de Villiers, 2007) predict False Belief success.

9 See Senghas et al.’s (2005) excellent description and analysis of the community structure and interaction patterns that catalyzed the emergence of Nicaraguan Sign Language, including the distinction between vertical (more mature/proficient model-to-learner) and horizontal (peer-to-peer) language input.
